# Ruptured Ectopic Pregnancy with Contralateral Ovarian Serous Cyst Adenoma Torsion: Laparoscopic Management of Double Trouble

**DOI:** 10.1155/2016/2980941

**Published:** 2016-10-20

**Authors:** Deepa Ganesh, Anirudh Rajkumar, J. S. Rajkumar, Venkatesan Guru

**Affiliations:** Department of Minimally Invasive Surgery, Lifeline Hospital, Chennai, Tamil Nadu, India

## Abstract

Adnexal torsion is responsible for 2.7% of all gynecological emergencies. Ectopic pregnancy is relatively common, occurring in 2% of all pregnancies. We report the second case of ruptured right tubal ectopic gestation with torsion of serous cystadenoma of left ovary. This was diagnosed after emergency laparoscopy done for acute abdomen. Right salpingectomy and left ovarian detorsion followed by cystectomy were done laparoscopically.

## 1. Introduction

Both ectopic pregnancy and adnexal torsion represent surgical emergencies. We hereby report the second case of ruptured ectopic pregnancy with coexisting contralateral ovarian torsion due to serous cystadenoma. The first reported case was in USA in 2008 [1]. Traditional management of salpingo-oophorectomy in adnexal torsion can destroy the future fertility in cases where the contralateral adexum is also diseased. The association between detorsion and systemic thrombosis is not certain. The management of this surgical emergency can be either laparotomy or laparoscopy. Minimally invasive surgery is a better option when the patient is hemodynamically stable as it has advantages of less pain, less blood loss, shorter hospital stay, and faster recovery.

## 2. Case Report

**Figure 1:**
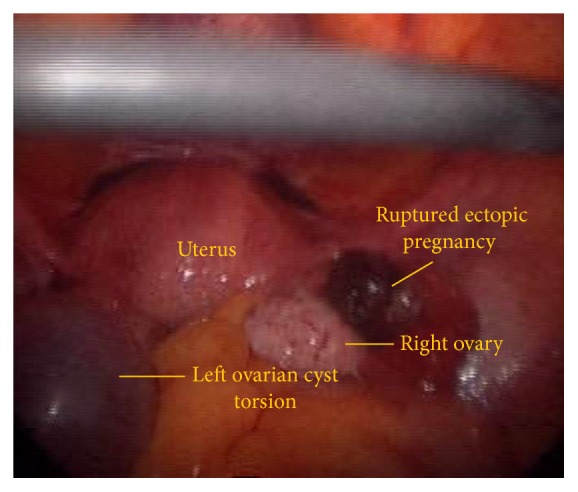
Right ruptured tubal gestation with left ovarian cyst torsion.

A 23-year-old lady married for six months presented with acute pain in the abdomen for the past 3 hours. She had one-month amenorrhea. There was severe tenderness in both right and left iliac fossa. On per speculum examination, minimal bleeding through os was seen and per vaginal examination the uterus was anteverted and of normal size with cervical motion tenderness. The patient had come with a sonography which was done elsewhere two weeks earlier, showing two 3 × 3 cm clear ovarian cysts. Urine pregnancy test was positive. Her haemoglobin was 11 gm and other laboratory investigations, total leucocyte count, differential leucocyte count, and urine, were found to be normal. Since the patient was hemodynamically stable, we proceeded with diagnostic laparoscopy under general anesthesia which revealed (1) right ruptured tubal ectopic pregnancy, (2) left ovarian cyst torsion, (3) normal right ovary, left tube, and uterine contour, and (4) haemoperitoneum of 50 cc. (Figure 1). Right salpingectomy with detorsion of left ovary followed by cystectomy was done. Intraoperative Doppler showed normal flow in left adnexa with normal appearance. Postoperative recovery was good. Histopathology revealed chorionic villi in right tube and serous cystadenoma of left ovary.

## 3. Discussion

Adnexal torsion accounts for 2.7% of all gynecological emergencies [2]. The twisting of an ovary or tube on its ligamentous support is called as adnexal torsion. It was initially described in American literature around 100 years ago by J. Bland Sutton. The school of teaching was to perform a salpingo-oophorectomy without untwisting to prevent a possible systemic thromboembolism from thromboses of ovarian vessels [3]. However the association between ovarian detorsion and systemic thromboembolism was never certain [4, 5]. About 20 years ago, Mage et al. demonstrated that detorsion helps in preservation of ovarian function [6]. Rodent models of adnexal recovery after 24–48 hours of surgical torsion were studied histologically. It was found that color, size, and edema of twisted adnexa cannot estimate the degree of necrosis. The blue-black appearance of ovarian tissue was due to initial lymphatic and venous stasis rather than significant arterial ischemia [7]. Gradually, long-term follow-up data of ovarian detorsion with minimal invasive surgery became available. Pre- and postoperative Doppler color flow and 3D pelvic ultrasound studies compared presence or absence of blood flow around ovary to demonstrate the recovered vascularity of ovarian tissue. Absence of blood flow around the ovary is highly specific but has low sensitivity. So, the presence of blood flow should not exclude a diagnosis of ovarian torsion when there is high clinical suspicion [8]. Normal ovarian morphology and follicular development were documented subsequently with 3D Doppler studies of ovarian volume and vascularity [9–11].This was followed by the three laparoscopic principals of prevention of recurrent torsion. The triplication of tubo-ovarian ligament, oophoropexy, and reduction of ovarian mass and volume with cystectomy and cyst aspiration were advocated [12, 13]. Ovariopexy refers to anchoring the ovary behind the uterus while oophoropexy refers to the anchoring of uteroovarian ligament behind the uterus. Removal of injured organ is advocated only when there is obvious ovarian gangrene or ligament disruption. The use of single port laparoscopy to untwist the ovary and ovarian cystectomy through the same single port encompasses the newer developments [13–15]. As in our patient, the other studies have also concluded that a conservative approach of untwisting the adnexa and salvaging the ovary via laparoscopy should be considered in adnexal torsion in reproductive age group when the time of onset of symptoms to surgery does not exceed 44 hours regardless of color and number of twists [16]. The intraoperative confirmation of return of normal vascularity with ovarian Doppler after laparoscopic detorsion is required. The high magnification of laparoscopic cameras helps to identify the necrosed tissue easily. At the time of surgery only the apparent gangrenous and thrombosed ovarian tissue and tubes need to be excised. This will comprise the minimally invasive fertility sparing surgery in young women.

This rare presentation highlights the importance of minimally invasive surgical technology in fertility sparing surgery in young women, with complex double procedures like ovarian detorsion, ovarian cystectomy, and salpingectomy all being done simultaneously, with relatively pain-free postoperative period.
